# Transcriptome Analysis on Chinese Shrimp *Fenneropenaeus chinensis* during WSSV Acute Infection

**DOI:** 10.1371/journal.pone.0058627

**Published:** 2013-03-19

**Authors:** Shihao Li, Xiaojun Zhang, Zheng Sun, Fuhua Li, Jianhai Xiang

**Affiliations:** Key Laboratory of Experimental Marine Biology, Institute of Oceanology, Chinese Academy of Sciences, Qingdao, China; Uppsala University, Sweden

## Abstract

Previous studies have discovered a lot of immune-related genes responding to white spot syndrome virus (WSSV) infection in crustacean. However, little information is available in relation to underlying mechanisms of host responses during the WSSV acute infection stage in naturally infected shrimp. In this study, we employed next-generation sequencing and bioinformatic techniques to observe the transcriptome differences of the shrimp between latent infection stage and acute infection stage. A total of 64,188,426 Illumina reads, including 31,685,758 reads from the latent infection group and 32,502,668 reads from the acute infection group, were generated and assembled into 46,676 unigenes (mean length: 676 bp; range: 200–15,094 bp). Approximately 24,000 peptides were predicted and classified based on homology searches, gene ontology, clusters of orthologous groups of proteins, and biological pathway mapping. Among which, 805 differentially expressed genes were identified and categorized into 11 groups based on their possible function. Genes in the Toll and IMD pathways, the Ras-activated endocytosis process, the RNA interference pathway, anti-lipopolysaccharide factors and many other genes, were found to be activated in shrimp from latent infection stage to acute infection stage. The anti-bacterially proPO-activating cascade was firstly uncovered to be probably participated in antiviral process. These genes contain not only members playing function in host defense against WSSV, but also genes utilized by WSSV for its rapid proliferation. In addition, the transcriptome data provides detail information for identifying novel genes in absence of the genome database of shrimp.

## Introduction

White spot syndrome (WSS), which is caused by white spot syndrome virus (WSSV), is one of the most dangerous diseases resulting in 90–100% mortality of shrimp [1]. Due to the serious impact of WSS on shrimp aquaculture, it is urgent to understand to the mechanisms involved in WSSV pathogenesis in shrimp.

To uncover the underlying mechanisms, high throughput approaches have been used to identify genes responding to WSSV infection. These includes cDNA microarray [2]–[4], suppression subtractive hybridization [5], SSH combining with differential hybridization [6], ESTs [7] and so on. A plenty of WSSV-modulated genes have been isolated, which contributes a lot to understanding the molecular mechanisms of host immune response to WSSV and developing possible antiviral technologies. Studies on specific genes further unveil their functions during WSSV-host interaction process. Many components in the Toll pathway, IMD pathway and JAK-STAT pathway can be stimulated by WSSV challenge, such as Toll [8], Spätzle [9], Pelle [10], TRAF6 [11], Dorsal [12], [13], Relish [14], [15], STAT [16], [17] *etc*. The Toll and IMD signaling pathways regulate expression of various antimicrobial peptides, which are important components of host humoral immunity [18], [19]. And the JAK-STAT pathway functions in antiviral defense [20]. Meanwhile, apoptosis and phagocytosis play key roles during WSSV-host interaction [21]. The above data has provided a preliminary description of host responses against WSSV infection in shrimp.

To our knowledge, most of the samples used for studying the above-mentioned genes were infected through artificial injection of WSSV. Although control groups were always set to eliminate irrelevant influences in the WSSV challenge experiments, manual operation might lead to other anonymous host responses. Another aspect arousing our attention was that individuals of the control group in those experiments were mostly supposed to be WSSV-free. However, during shrimp culture, the distribution of WSSV was prevalent and animals carrying WSSV could normally survive [22]–[25]. Quantitative analysis of WSSV infection in *Penaeus monodon* revealed that WSSV pathogenesis experienced three stages, including eclipse, logarithmic and plateau, in accordance with light, moderate and heavy infection stages of the shrimp [26]. Some genes isolated from WSSV were considered to be important for WSSV latent infection (LI) to the host [27]. One of the genes, ORF89, was likely to inhibit WSSV replication and maintain the latency state through repressing expression of a protein kinase and the thymidine-thymidylate kinase genes of WSSV [28]. Other studies further demonstrated that acute outbreak of WSS from LI stage could be caused by rapid changes of environmental factors, such as salinity [29] and temperature [30]. Understanding on the molecular mechanisms regulating the acute infection (AI) will provide useful information for developing antiviral technologies.

Although a lot of genes have been verified to be related with host responses against bacteria and virus, no report was noticed to illustrate whether they worked in the AI stage and what genes were involved in the AI stage. In the present study, we ignored the influence of environmental factors and only focused on the differences of host responses between LI shrimp and AI shrimp. We selected naturally infected shrimp in LI and AI stages as experimental materials. This could also eliminate the impact of manual operation on the experimental animals. The next-generation sequencing and bioinformatic techniques were applied to compare the transcriptome difference of LI shrimp and AI shrimp.

## Materials and Methods

### Preparation of shrimp materials

WSSV-carrying shrimp *F. chinensis*, obtained from a local shrimp farm, were reared in 8 m^3^ fiberglass tanks and fed with artificial food pellets. After appearance of WSS in a few animals, the shrimp without symptom were collected individually. The cephalothoraxes and pleopods of the collected shrimp were dissected and preserved in liquid nitrogen until RNA and DNA extraction.

According to our previous data [31], WSSV experienced a rapid proliferation stage in tested *F. chinensis*, beginning with a virus copy number of about 4.16×10^3^/ng pleopods DNA and reached to a steadily replication stage with a virus copy number of about 6.80×10^5^/ng pleopods DNA. In the present study, shrimp carrying an amount of WSSV between the two values was considered as AI shrimp, while shrimp carrying less WSSV copies was regarded as LI shrimp. To obtain shrimp in LI and AI stages, WSSV copy number in the pleopods was quantitatively analyzed according to the method described by You *et al.* (2010) with slight modification [32]. Briefly, DNA was extracted from pleopods using the Axyprep Multisource Genomic DNA Miniprep Kit (Axygen, USA) following recommended protocols. A 281 bp fragment of the WSSV VP28 gene amplified by the primers VP28F1 and VP28R1 (Table S1). The fragment was cloned into pMD-19 T simple cloning vector (TaKaRa, Japan). The plasmid was then extracted and quantified, and the copy number was calculated. Standard curves were constructed using 10-fold dilutions of the plasmid DNA ranging from 10^8^ to 10^3^. Based on the 281 bp fragment, another pair of primers VP28F2 and VP28R2 (Table S1) amplifying a 141 bp fragment was designed. SYBR Green-based quantitative RT-PCR (qPCR) was performed by Eppendorf Mastercycler® ep realplex (Eppendorf, Germany) in diluted plasmid and pleopods DNA under the conditions described below: denaturation at 94°C for 2 min; 40 cycles of 94°C for 20 s, 55°C for 20 s, and 72°C for 20 s. WSSV copy number in per ng pleopods DNA was calculated based on the standard curve. Based on the WSSV copy number [31], 10 individuals (length/cm: 7.99 ± 0.69; weight/g: 6.33 ± 1.67) were deemed as LI shrimp and another 10 individuals (length/cm: 8.35 ± 0.56; weight/g: 7.18 ± 1.56) as AI shrimp, respectively. Their cephalothoraxes were used for transcriptome analysis.

### RNA isolation and Illumina sequencing

The paired-end RNA-seq method [33] was performed to sequence the transcriptome of LI shrimp and AI shrimp. In brief, total RNA was extracted from *F. chinensis* (n = 10 for each group) using Unizol reagent (UnionGene, China) and treated with DNase I. RNA amounts were estimated spectrophotometrically (NanoDrop Technologies). Polyadenylated (polyA+) RNA was purified from total RNA using Sera-mag oligo(dT) beads, fragmented to a length of 100–500 bases, reverse transcribed using random hexamers, end repaired and adaptor-ligated, according to the manufacturer’s protocol (Illumina). Ligated products of 300–500 bp were excised from agarose and PCR-amplified (15cycles). Products were cleaned using a MinElute column (Qiagen) and paired-end sequenced on a Genome Analyzer II (Illumina), according to manufacturer’s instructions.

### Bioinformatic analyses

The 90 bp + 90 bp paired-end sequences generated from the non-normalized cDNA library representing the LI and AI shrimp of *F. chinensis* were assembled using RNA-Seq De novo Assembly program Trinity [34], followed by TIGR Gene Indices clustering tools (TGICL) [35], with default parameters. Adapter sequences and sequences with suboptimal read quality (i.e., PHRED score of 32.0) were eliminated. The raw sequence reads from the cDNA library were then mapped to the non-redundant sequence data using Burrows-Wheeler Aligner (BWA) (http://bio-bwa.sourceforge.net/). In brief, raw reads were aligned to the assembled, non-redundant transcriptomic data, such that each read was mapped to a unique transcript. To provide a relative assessment of transcript-abundance, the numbers of raw reads that mapped to individual contigs were normalized for sequence length (i.e., Fragments Per Kilobase of transcript per Million mapped reads, FPKM [36]).

The non-redundant transcriptomic dataset for *F. chinensis* was then analyzed using an established approach. Briefly, assembled contigs were firstly annotated (using BLASTn and BLASTx algorithms) with sequences available in public NCBI (www.ncbi.nlm.nih.gov) database. Proteins were conceptually translated from the open reading frames (ORFs) of individual sequences using ESTScan. Protein-coding sequences were classified functionally using InterProScan, employing the default search parameters. Based on their homology to conserved domains and protein families, proteins predicted for *F. chinensis* were assigned parental (i.e., level 2) Gene Ontology (GO) terms (http://www.geneontology.org/). Deduced proteins with homologues in other organisms were used to determine the Clusters of Orthologous Groups of proteins (COG) item [37] and mapped to conserved biological pathways utilizing the Kyoto Encyclopedia of Genes and Genomes (KEGG) [38].

Differentially expressed genes (DEGs) were obtained based on the FPKM of the genes in LI and AI groups, followed by a multiple hypothesis testing, False Discovery Rate (FDR) control [39], to correct for p-value. The FPKM of a certain gene was calculated in LI group and AI group. The gene with a FPKM ratio lager than 2 or smaller than 0.5 and with a FDR≤0.001 was considered as differentially expressed gene. Pathway analysis on the DEGs was also performed using KEGG. Annotated pathways with a Q value<0.05 were regarded as differentially expressed pathways.

ALF and PO amino acid sequences of other species (Table S2) were obtained from the NCBI website and the sequence alignment was produced with online ClustalW2 software (http://www.ebi.ac.uk/Tools/msa/clustalw2/). Phylogenic tree was constructed using MEGA software version 4.0 [40].

### qPCR analysis of selected genes from the transcriptome

Fourteen genes, including 7 genes encoding for anti-lipopolysaccharide factors (ALF), 3 genes encoding for prophenoloxidase (proPO) and 4 other unigenes, were selected to detect their expression levels in LI and AI shrimp by qPCR. qPCR was performed following the above-mentioned methods with modified annealing temperature for each pair of primers. The primers’ information was listed in Table S1. The data obtained from qPCR were analyzed for statistical significance using Graph-Pad Prism [41]. The significance at *P*<0.05 was analyzed using one-way ANOVA. The qPCR result was then compared with transcriptome data to detect the expression correlation of each gene.

## Results and Discussion

### Validation of the LI and AI shrimp based on the WSSV copy number

The WSSV copy number was (2.46±0.23)×10^3^/ng pleopods DNA in the selected LI shrimp (n = 10), and was (2.93±1.37)×10^5^/ng pleopods DNA in the selected AI shrimp (n = 10). The results showed that the selected LI and AI shrimp fit for the transcriptome analysis.

### Transcriptome sequences assembly and analysis

A total of 64,188,426 Illumina reads, including 31,685,758 reads from the LI group and 32,502,668 reads from the AI group, were produced from the Chinese shrimp, *F. chinensis*. The detail sequence information of the transcriptome was listed in Table 1. Generally, 46,214 unigenes (mean length: 512 bp; range: 150–15,094 bp) and 52,658 unigenes (mean length: 573 bp; range: 150–14,194 bp) were assembled in the LI group and AI group, and 46,676 unigenes (mean length: 676 bp; range: 200–15,094 bp) were yielded in the merged group. About 86.1% and 85.5% of the raw reads from LI group and AI group, and 85.8% of the total raw reads from the merged group, were re-mapped to the assembled unigenes. The N50 for the LI group, AI group and merged group were 693 bp, 852 bp and 982 bp, respectively.

**Table 1:** General information of the transcriptome from LI and AI shrimp.

Dataset name	LI	AI	All
Total raw reads (paired-end)	31,685,758	32,502,668	64,188,426
Total clean reads	26,060,240	27,057,642	53,117,882
Q20 percentage	95.03	94.98	95.00
N percentage	0	0	0
GC percentage	48.55	48.16	48.35
Unigenes	46,214	52,658	46,676
Raw reads mapped to unigenes (%)	86.1	85.5	85.8
Mean length (bp)	512	573	676
N50 (bp)	693	852	982
Min-Max length (bp)	150-15094	150-14194	200-15094

**Figure 1:**
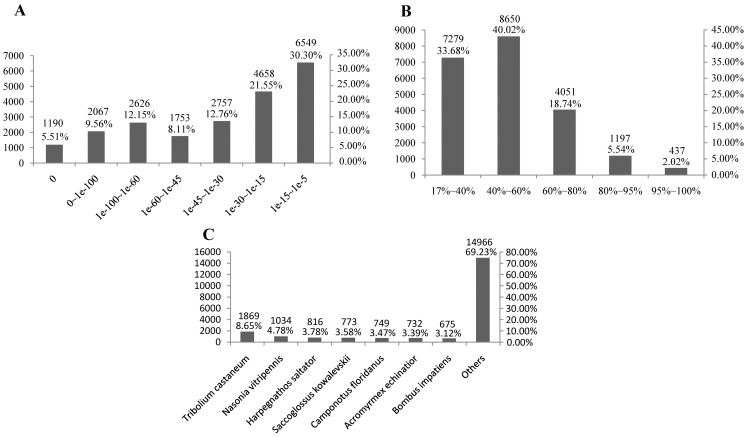
Characteristics of homology search of the assembled unigenes against the nr database. (A) E-value distribution of BLAST hits for each unique sequence with a cut-off E-value of 1.0E^-5^. (B) Similarity distribution of the top BLAST hits for each sequence. (C) Species distribution is shown as a percentage of the total homologous sequences with an E-value of at least 1.0E^-5^. The first hit of each sequence was used for analysis.

Before analysis of differentially expressed genes which responded to WSSV acute infection, an elementary sequence analysis was carried out to comprehend the shrimp transcriptome based on the unigenes from the merged group. After a homology search in the non-redundant protein database at NCBI, a total of 21,600 unigenes, which took up a proportion of 46.28% in all the unigenes, showed significant BlastX hits of known protein sequences (e-value cut-off: <10^-5^). The distribution of significant BlastX hits over different organisms was also analyzed. Due to the lack of genomic information in shrimp, the majority of the assembled sequences (1869 sequences, 8.65%) matched genes from *Tribolium castaneum* (Figure 1).

### Functional annotation of all unigenes

**Figure 2:**
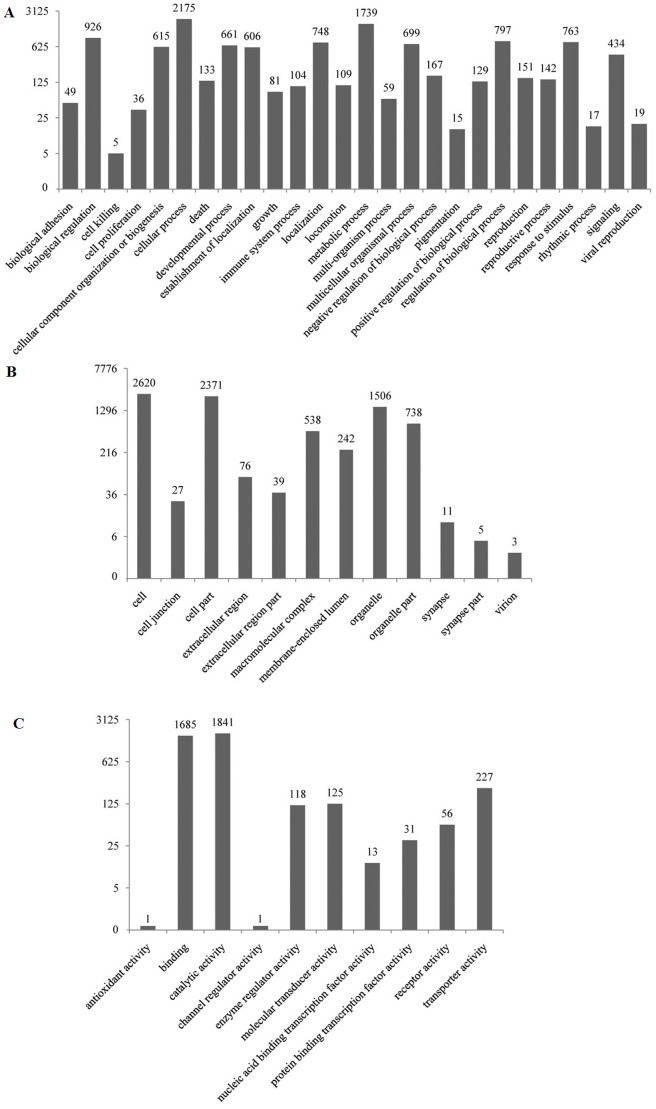
GO annotations of unigenes from the merged database of transcriptome from shrimp at LI and AI stages. Most non-redundant sequences can be divided into three major categories, including biological process (A), cellular component (B), and molecular function (C).

Further sequence annotation was first performed on the unigenes from the merged group. The putative functions of all unigenes were analyzed based on GO and COG classifications. A total of 4,101 unigenes had GO annotations. 2,815 unigenes were mapped to biological processes, 2,710 unigenes were mapped to cellular components, and 2,881 unigenes were mapped to molecular functions. Most of the biological process related genes were involved in cellular process, metabolic process and biological regulation. Most of the cellular component related genes were involved in cell, cell part and organelle. Most of the molecular function related genes were involved in catalytic activity, binding and transporter activity (Figure 2).

**Figure 3:**
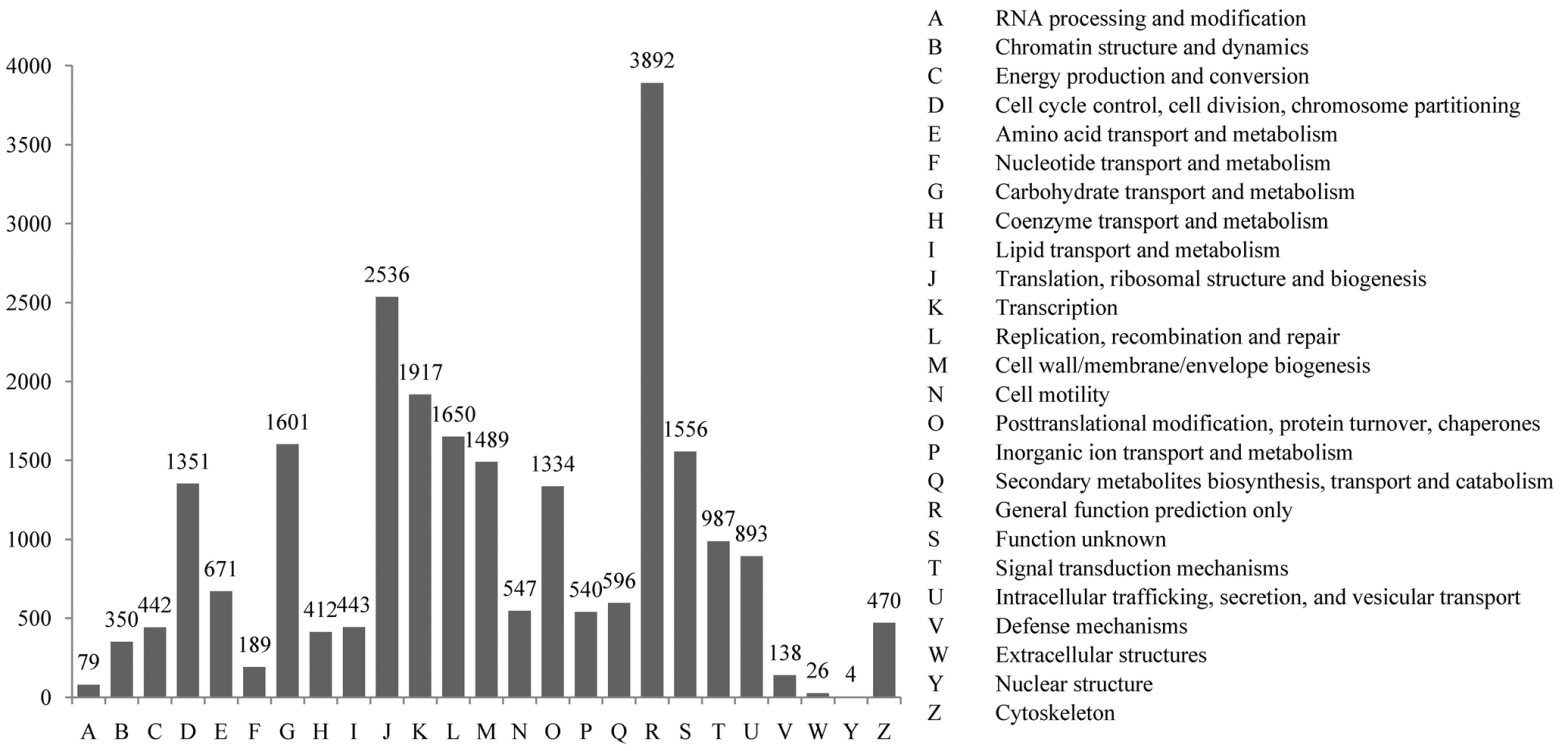
COG classification of unigenes from the merged database of transcriptome from shrimp at LI and AI stages.

COG classification of the unigenes is important for functional annotation and evolutionary studies [42]. A total of 8,575 unigenes were finally mapped on 25 different COG categories (Figure 3). The largest COG group was “general function predicted only” (3,892 unigenes),, followed by “translation, ribosomal structure and biogenesis” (2,536 unigenes) and “transcription” (1,917 unigenes).

In order to obtain more information for predicted functions of the unigenes, the genes from the merged group were categorized in the KEGG database. A total of 16,232 unigenes were classified into 239 KEGG pathways. A lot of immune related pathways, including phagosome, lysosome, extracellular matrix (ECM)-receptor interaction, Fc gamma R-mediated phagocytosis, leukocyte trans-endothelial migration, complement and coagulation cascades, and many signaling transduction pathways such as mitogen-activated protein kinase (MAPK) signaling pathway, vascular endothelial growth factor (VEGF) signaling pathway, JAK-STAT signaling pathway, peroxisome proliferator-activated receptors (PPAR) signaling pathway, Toll-like receptor signaling pathway and so on, were predicted in the KEGG database (Table S3).

### Differentially expressed genes

**Figure 4:**
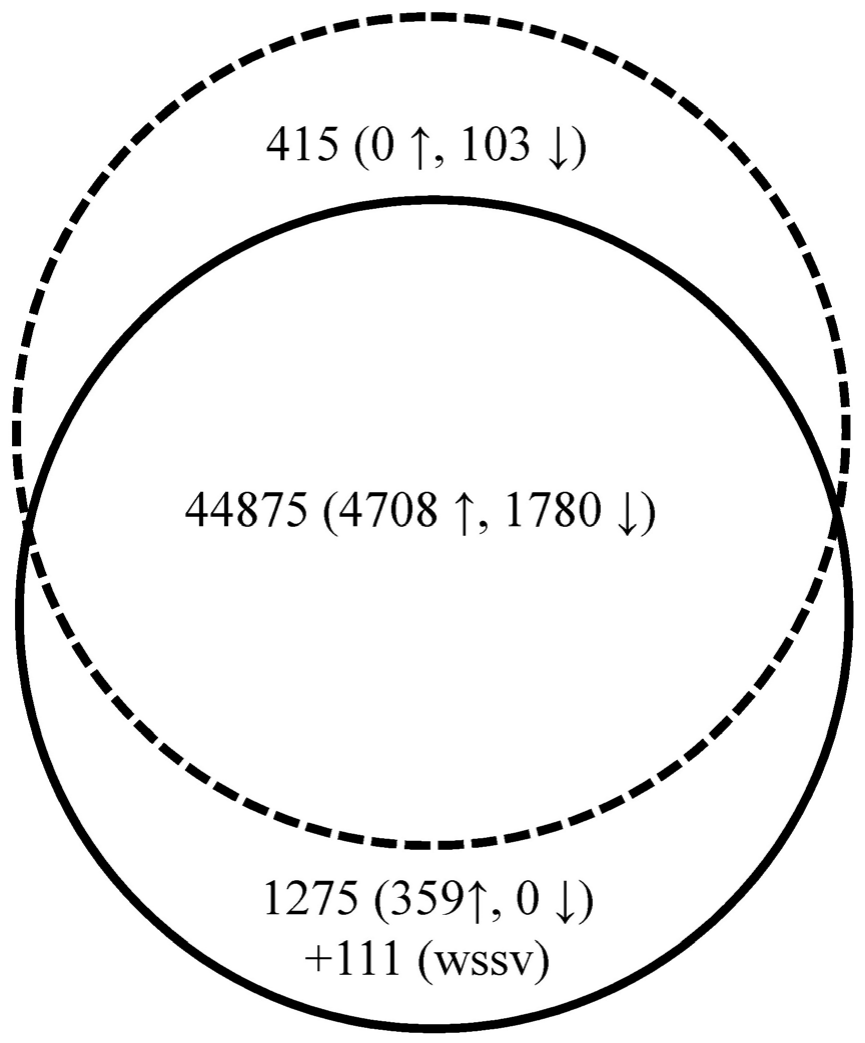
Differentially expressed genes in LI and AI shrimp. The dotted circle represented DEGs in LI shrimp and the solid circle represented DEGs in AI shrimp. Gene number was shown with up arrow and down arrow representing up-regulation and down-regulation of these genes in the AI shrimp, respectively. Mutual genes in the transcriptome data of LI and AI shrimp were closed in the area cross two circles, while specific genes were in single circle. The gene number from WSSV was added additionally.

Primary sequence analysis and annotation on all unigenes in the merged group provided us much useful information to understand the transcriptome and further to analyze DEGs that induced by WSSV acute infection. As shown in Figure 4, among the 46,676 unigenes, a total of 7,061 DEGs were screened after comparison between the LI group and AI group. 5,178 genes were found to be differentially up-regulated genes (DUGs) and 1,883 genes were identified as differentially down-regulated genes (DDGs) in AI group in comparison with LI group. Among these DEGs, 6,488 genes, including 4,708 DUGs and 1,780 DDGs, existed both in LI and AI group, 103 genes were only found in the LI group and 359 genes were specifically detected in the AI group. The data, including Gene ID, Gene Length, Raw fragments in LI/AI group, FRKM in LI/AI group, log2(AI-FPKM/LI-FPKM), Fold Ratio (AI/LI) and annotation information, of all the DEGs were provided in Table S4. In addition, 111 nucleotide sequences that matched WSSV genome sequence (detail information in Table S5) were discovered in the AI group but no transcript was found in LI group, further indicating a rapid proliferation of WSSV in the AI group and the latency state of WSSV in the LI shrimp. KEGG analysis on the DEGs revealed that 64 pathways were significantly changed (Q value<0.05) in AI group compared with LI group. Some pathways regarding to shrimp immunity such as *Vibrio* cholerae infection, ECM-receptor interaction, phagosome, complement and coagulation cascades, Peroxisome proliferator-activated receptors (PPAR) signaling pathway etc., and to metabolic such as tyrosine metabolism, protein digestion and absorption, fat digestion and absorption, starch and sucrose metabolism etc., and to other biological processes, were found differentially expressed between LI and AI shrimp. These data indicated comprehensive changes of the shrimp physiological status during WSSV acute infection. The top 25 pathways based on the Q value were listed in Table 2 (for details of all differentially expressed pathways, see Table S3: #1–64).

**Table 2:** Top 25 differentially expressed pathways between LI and AI shrimp.

#	Pathway	Number of DEGs with pathway annotation	P-value	Q-value	Pathway ID
1	Amoebiasis	240	1.31E-19	3.12E-17	ko05146
2	Vibrio cholerae infection	195	8.43E-13	1.01E-10	ko05110
3	Metabolic pathways	520	9.60E-12	7.65E-10	ko01100
4	Tyrosine metabolism	63	2.83E-10	1.69E-08	ko00350
5	ECM-receptor interaction	106	1.34E-08	6.40E-07	ko04512
6	Protein digestion and absorption	87	3.04E-08	1.21E-06	ko04974
7	Riboflavin metabolism	24	1.22E-07	4.16E-06	ko00740
8	Staphylococcus aureus infection	43	1.44E-07	4.30E-06	ko05150
9	Glycine, serine and threonine metabolism	34	1.85E-07	4.91E-06	ko00260
10	Metabolism of xenobiotics by cytochrome P450	43	2.75E-07	6.58E-06	ko00980
11	Phagosome	115	3.32E-07	7.20E-06	ko04145
12	Lysosome	102	4.74E-07	9.45E-06	ko04142
13	Steroid hormone biosynthesis	32	2.21E-06	3.77E-05	ko00140
14	Fat digestion and absorption	32	2.21E-06	3.77E-05	ko04975
15	Phenylalanine metabolism	25	2.74E-06	4.37E-05	ko00360
16	beta-Alanine metabolism	23	4.67E-06	6.98E-05	ko00410
17	Starch and sucrose metabolism	41	5.20E-06	7.31E-05	ko00500
18	Complement and coagulation cascades	48	9.04E-06	1.20E-04	ko04610
19	Butanoate metabolism	38	1.78E-05	2.13E-04	ko00650
20	Galactose metabolism	31	1.84E-05	2.13E-04	ko00052
21	Retinol metabolism	29	1.88E-05	2.13E-04	ko00830
22	Drug metabolism - cytochrome P450	40	2.22E-05	2.41E-04	ko00982
23	Carbohydrate digestion and absorption	28	2.83E-05	2.94E-04	ko04973
24	Neuroactive ligand-receptor interaction	67	3.14E-05	3.13E-04	ko04080
25	Amino sugar and nucleotide sugar metabolism	52	7.64E-05	7.31E-04	ko00520

### Classification of immune-modulation related DEGs

**Figure 5:**
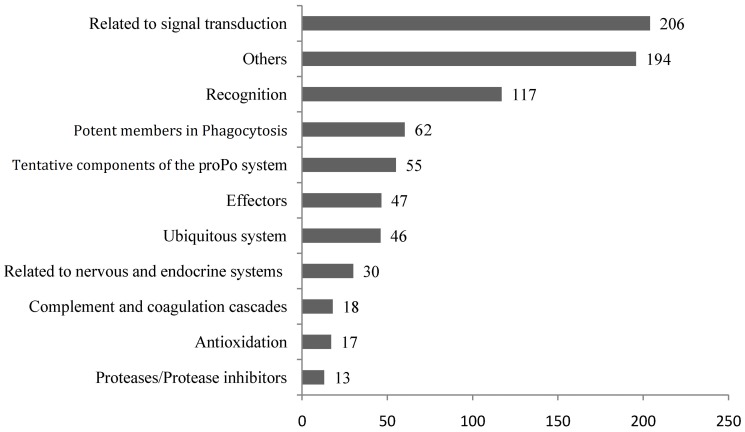
Classification of immune-modulation differentially expressed genes (DEGs). These genes were selected from all DEGs in the transcriptome of latent infection (LI) and acute infection (AI) shrimp.

Among the 7,061 DEGs, immune-related genes were mainly distributed in DUGs while DDGs were mainly metabolic related genes (data not shown). 805 DEGs were identified as immune-modulation genes after screening of the total DEGs (Table S6). These selected DEGs were categorized into 11 groups (Figure 5). Genes related to signal transduction (206) accounted for the maximum amount, followed by genes of non-classified (others, 194), recognition (117), potent members in Phagocytosis (62), tentative components of the proPO system (55), effectors (47), ubiquitous system (46), related to nervous and endocrine system (30), complement and coagulation cascades (18), antioxidation (17), and proteases and protease inhibitors (13).

### Up-regulation of genes in Toll and IMD signaling pathways and AMPs in AI shrimp

The well-studied signaling pathways involved in host innate immune response against pathogens are Toll pathway and the Immune deficiency (IMD) pathway, which activate the expression of antimicrobial peptide (AMP) genes and regulate the host humoral response [43]–[46]. Previous studies in *Drosophila melanogaster* revealed that Toll pathway and IMD pathway not only participated in anti-bacterial processes [47], [48], but also played important roles against viral infection [49], [50]. Many key components in Toll pathway and IMD pathway were identified from the transcriptome, including Spätzle, toll-like receptors, MyD88, tumor necrosis factor receptor-associated factor 6, dorsal, cactus, Ikkepsilon1 and relish (Table S6). These genes were all positively regulated in the AI shrimp, which kept consistent with previous studies and showed their contributions on regulating the expression of AMPs [8], [9], [11], [12], [18].

As downstream effectors of Toll and IMD pathways, different kinds of antimicrobial peptides, including ALFs, crustins, and other antimicrobial related peptides, were identified from the transcriptome data (Table S6). The transcriptome data showed that seven ALFs were all up-regulated in the AI shrimp.

The cDNA nucleotide sequence encoding ALF could generate a putative protein of approximately 125 amino acids, including a signal peptide of approximately 25 amino acid residues [51]. The mature ALF peptide is composed of a functional domain with three α-helices flanking a four-strand β-sheet [52]. The second and third β-strands are linked by a disulfide bond and form an amphipathic loop responsible for LPS-binding [52]. This cationic residues rich region exhibits activities against gram-negative bacteria and gram-positive bacteria [53]. ALFs have been isolated in many crustacean species. Some species generate different kinds of ALF peptides, whose transcription level can be up-regulated by gram-negative or gram-positive bacteria infection [54]-[58]. Moreover, the recombinant ALFs both from shrimp and crab are highly effective against gram-negative and/or gram-positive bacteria [55]–[57], [59]. In addition, previous studies also revealed that ALF in *Pacifastacus leniusculus* and *P. monodon* provided protection against WSSV infection [60], [61].

**Figure 6:**
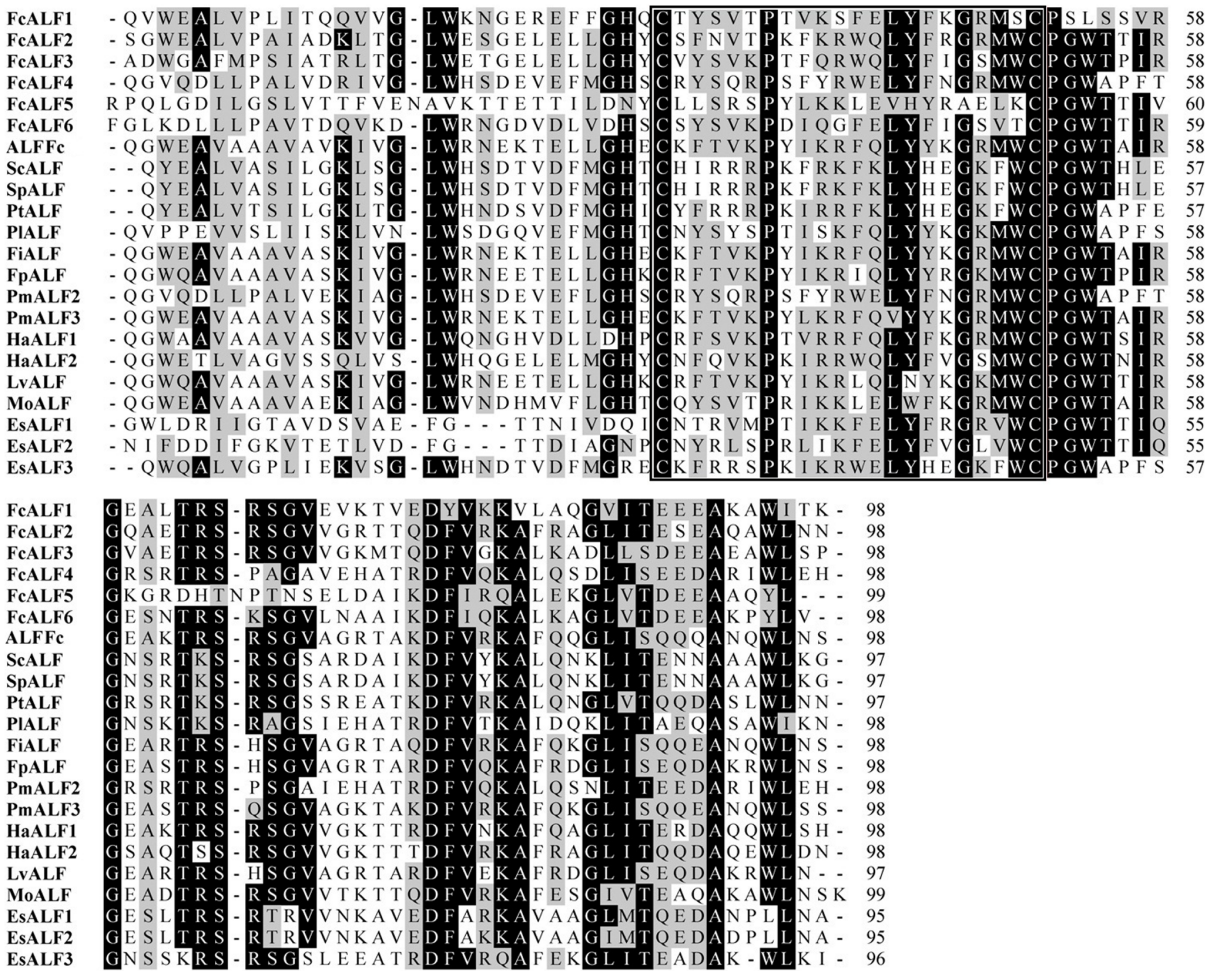
Multiple alignments of FcALF isoforms with ALF from other species. Sequence information were summarized in Table S2. Identical amino acid residues were highlighted in dark and similar amino acids were highlighted in gray. Inserts (–) were been added to maximize sequence identity. The putative LPS-binding domain was marked in a frame.

**Figure 7:**
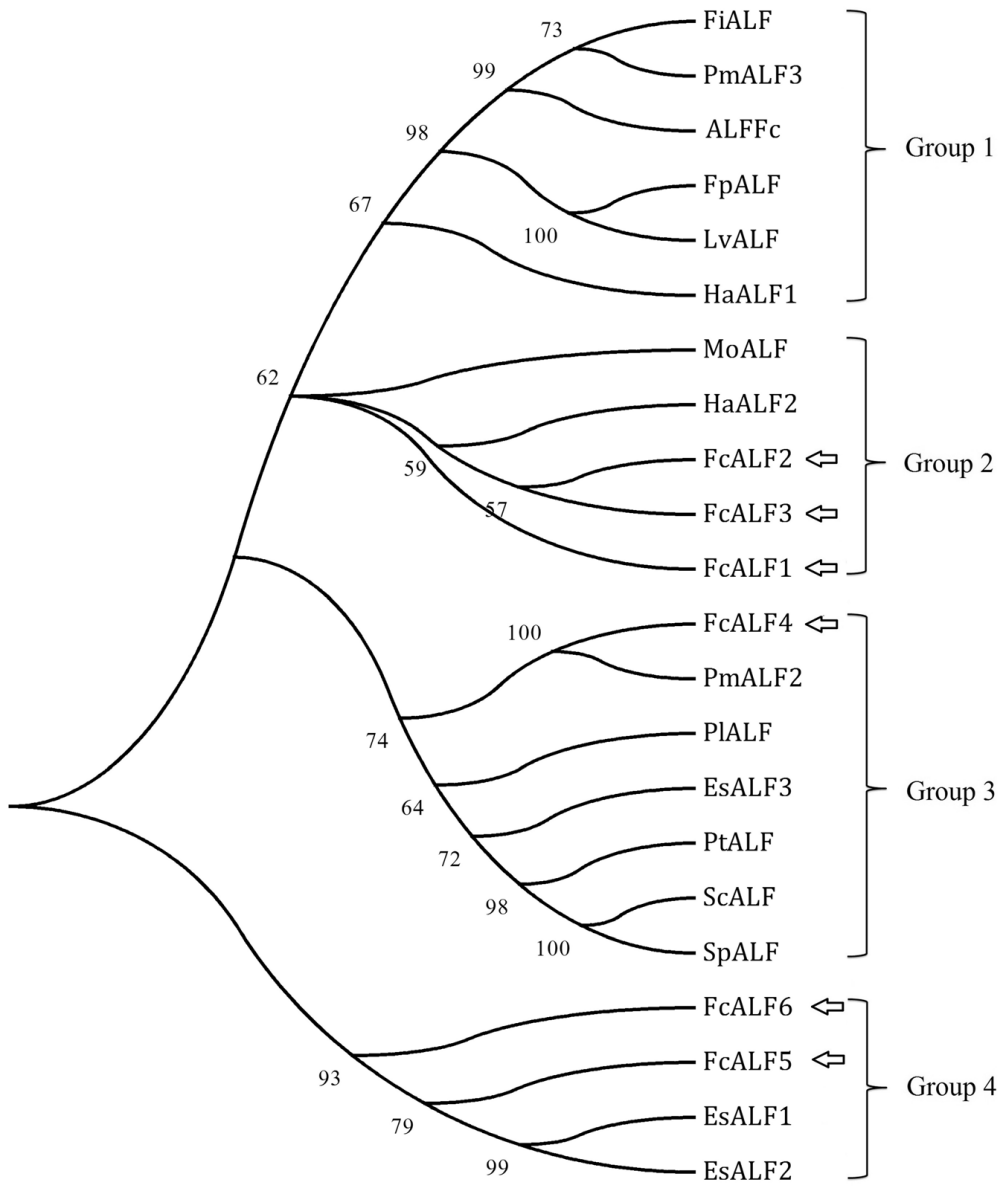
The neighbor-joining phylogenetic tree of FcALF isoforms and ALF homologues from other species. Sequence information were summarized in Table S2. Signal peptide of each ALF was deleted when analyzed. Bootstrap analysis of 1000 replicates was carried out to determine the confidence of tree branch positions. And the numbers marked on the tree branches represent the bootstrap values.

**Figure 8:**
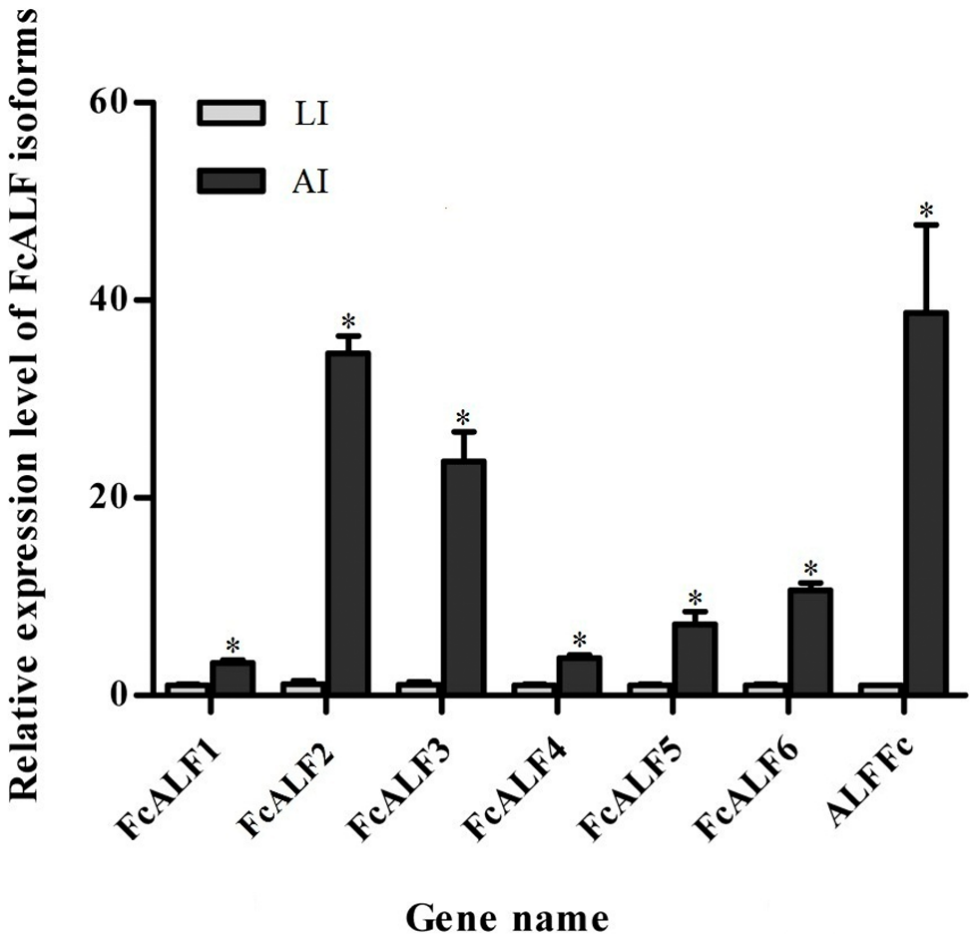
Expression patterns of FcALF isoforms in latent infection (LI) and acute infection (AI) shrimp. Data were expressed as the mean fold change (means±S.E., n  =  10). Statistical significance was calculated by Tukey multiple comparison tests and the Student’s *t-test*. Bars with different letters indicate statistical differences (*P*<0.05).

All the deduced ALF peptides derived from the transcriptome data shared the conserved LPS-binding domain when compared with ALFs from other species (Figure 6). One of them was previously reported as ALFFc and data showed that the expression of ALFFc could be stimulated by *Vibrio anguillarum* infection [62]. However, we still did not know whether ALFFc responded to WSSV infection. The other 6 ALFs (JX853774-JX853779) in the Chinese shrimp were identified as new isoforms, which were designated as FcALF1-6. Phylogenic analysis of ALFs from crustacean species showed that they were categorized into 4 groups (Figure 7). ALFFc was classified into group 1, FcALF1-3 was classified into group 2, FcALF4 was in group 3, and FcALF5 and FcALF6 were in group 4. qPCR analysis on the expression patterns of different variants of ALFs in the LI shrimp and AI shrimp was carried out. All of them were up-regulated in AI shrimp compared to LI shrimp, while distinct ALFs responded differently (Figure 8). ALFFc, FcALF2 and FcALF3 exhibited much higher up-regulated expression level than other types. Different responses of ALFs to different pathogens were also reported in *H. americanus*, in which HaALF1 was stimulated by *Vibrio fluvialis* challenge, while HaALF2 was not affected [63]. However, the responsive model of ALFs to pathogens in the Chinese shrimp should be described in detail in future studies.

### Ras-regulated endocytosis during WSSV infection

The Ras superfamily is a group of small GTPases exhibiting high-affinity binding for GDP and GTP, which can be divided into five families, the Ras family, the Rho family, the Rab family, the Ran family and the Arf family, based on their structure and function [64]. The small GTPases exert their function with the help of GTPase Activating Proteins (GAPs) to enhance their intrinsic GTPase activity and Guanine Exchange Factors (GEFs) to exchange bound GDP for GTP [65]. By regulating actin and myosin recruitment, these GTPases are essential for phagocytosis, a form of endocytosis [66]–[70].

A number of genes with annotation of Ras, Rab, Rap, Rho, GAP, GEF, dynamin, myosin, clathrin, and actin were up-regulated in the AI shrimp (Table S6), indicating that Ras-regulated phagocytosis participated in the process of WSSV rapid proliferation stage. Previous studies demonstrated that the Ras-related phagocytosis was involved in the host-virus interaction. The Rab GTPase from *Marsupenaeus japonicus* could regulate shrimp hemocytic phagocytosis to offend WSSV via a protein complex consisting of the Rab, beta-actin and tropomyosin of the shrimp, and envelope protein VP466 of WSSV [71]. Meanwhile, the Rab GTPase-regulated phagocytosis also contributed to anti-bacterial effect [72]. In addition, a Ran isoform was proved to be obviously up-regulated in shrimp after WSSV-challenge [73] and studies showed that it possessed a similar function on hemocytic phagocytosis by interaction with myosin in antiviral shrimp [67]. Although the above evidence revealed the antiviral function by Ras-regulated phagocytosis, studies also indicated virus could enter the host cells via clathrin-dependent endocytosis [74], [75], which was mediated by Ras-phosphoinositide 3-kinase (PI3K) signaling pathway [76] and dynamin [77]. Another Rab (PmRab7) in shrimp was shown binding to the WSSV structure protein VP28 and conducive to WSSV infection [78]. These data indicated that Ras superfamily members and Ras-mediated endocytosis might perform more complicated function except for specified antiviral defense or a WSSV-infection assistant during host-virus interaction.

### The proPO-activating system responds to WSSV acute infection

The proPO-activating system mainly includes genes such as kinds of serine proteinases and their inhibitors (serpins), prophenoloxidase-activating enzyme (PPA), proPO and its active form, phenoloxidase (PO) [79]. The activation of the proPO-activating system is switched on by lipopolysaccharides (LPS), β-1,3-glucans or peptidoglycans (PG) [80], [81], which are the important cell-wall components of bacteria or fungi. After stimulated by LPS, β-1,3-glucans or PG, a serine proteinase cascade is first triggered [82], which leads to the cleavage of the pro-form of the prophenoloxidase-activating enzyme (pro-PPA) into active PPA. Then PPA further activates the proPO into the active enzyme, PO, through proteolysis of its pro-peptide [79]. Serine proteinase inhibitors or serpins negatively regulated proPO activation via specific inhibition of the cascades components [83]. The proPO-activating system participates in host defense in arthropods by enhancing phagocytosis, initiating nodule or capsule formation, mediating coagulation and producing fungistatic substances [79].

In the present study, 55 DEGs were annotated to be tentative members of the proPO-activating system (Table S6). These genes were mainly kinds of serine proteinases, including clip domain serine proteinase, serine protease-like protein, chymotrypsin-like serine protease, and their inhibitors such as serpin, serpin peptidase inhibitor and pacifastin. The PPA gene and proPO gene were also identified in the transcriptome. Studies have revealed that this system can be positively activated by bacteria invasion or fungi infection [84]–[88], while less evidence displayed its function against virus infection. The expression level of proPO transcripts and the enzyme activity of PO in WSSV-injected crayfish stayed the same with that in sham-injected crayfish; therefore a hypothesis that WSSV inhibited the proPO system upstream of phenoloxidase or simply consumed the native substrate for the enzyme was proposed [89]. However, a reactive component 5,6-dihydroxyindole (DHI), which is generated by PO, not only has the antibacterial and antifungal activities, but also has strong toxicity against virus pathogen [90], indicating the involvement of the proPO-activating system in the antiviral immune defense.

The present transcriptome data revealed that most members in the serine proteinase cascade and proPO system responded to WSSV rapid proliferation in *F. chinensis* (Table S6). Serine proteases were all up-regulated in AI shrimp, showing a positive response of the serine proteinase cascade in the antiviral immune defense. Unexpectedly, serpins were also up-regulated in the WSSV-infected shrimp, which seemed contrary to the positive response of serine proteinases. A similar consequence was shown by the PPAs and proPOs. Two different PPAs were identified in the transcriptome. One of them, which was annotated as prophenoloxidase activating enzyme III, was up-regulated in the AI shrimp. Whereas the other one, annotated as prophenoloxidase-activating enzyme 1a, was down-regulated (Table S4). It might be the reason that an auto-modulation of the proPO-activating system exists during host immune defense to avoid damage of host tissues and cells by excess reactive components generated by PO [90].

**Figure 9:**
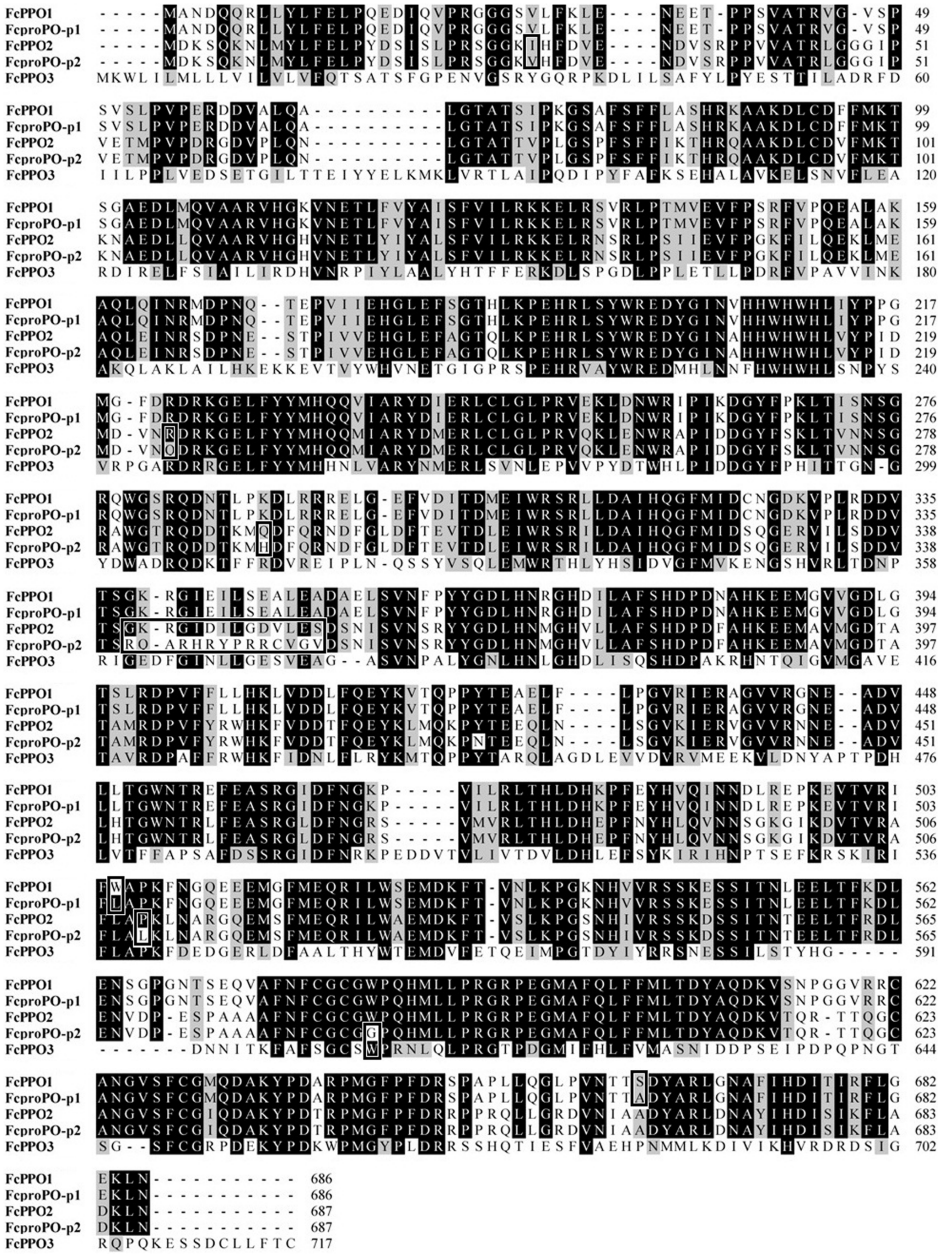
Multiple alignments of all known proPO isoforms from the Chinese shrimp. Sequence information were summarized in Table S2. Identical amino acid residues were highlighted in dark and similar amino acids were highlighted in gray. Inserts (–) were added to maximize sequence identity. Differences between deduced proPO amino acid sequences in the present study and published version was shown in frames.

**Figure 10:**
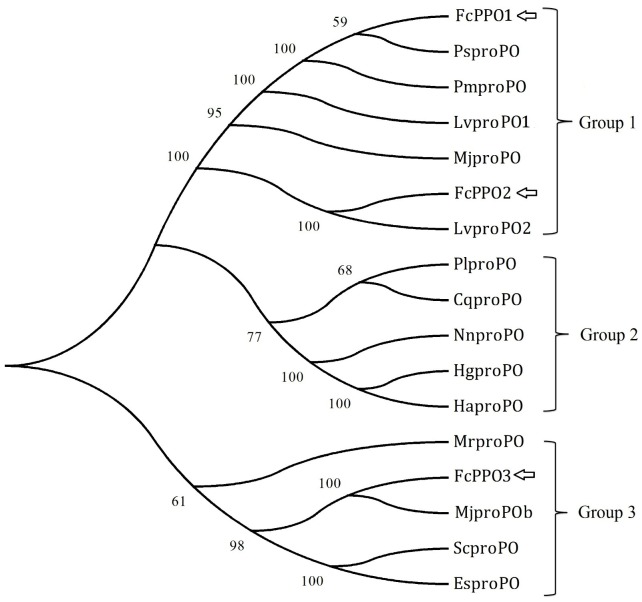
The neighbor-joining phylogenetic tree of FcproPO isoforms and proPO homologues from other species. Sequence information was summarized in Table S2. Bootstrap analysis of 1000 replicates was carried out to determine the confidence of tree branch positions. And the numbers marked on the tree branches represent the bootstrap values.

**Figure 11:**
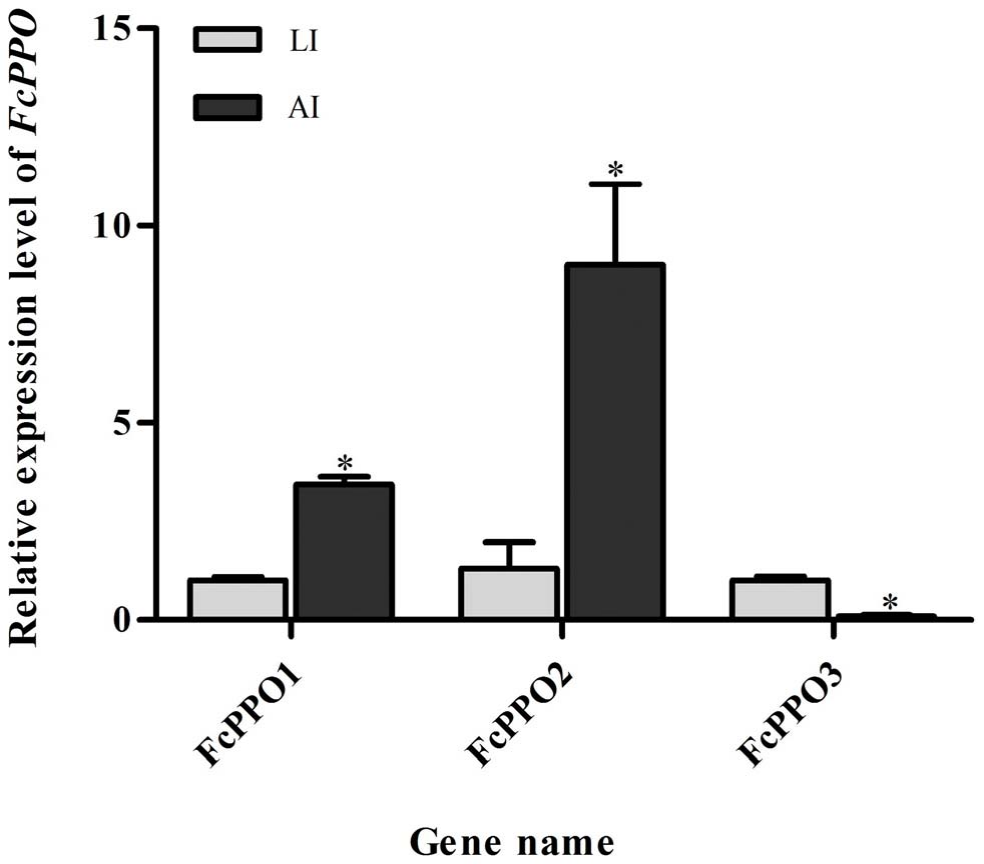
Expression patterns of FcproPO isoforms in latent infection (LI) and acute infection (AI) shrimp. Data were expressed as the mean fold change (means±S.E., n  =  10). Statistical significance was calculated by Tukey multiple comparison tests and the Student’s *t-test*. Bars with different letters indicate statistical differences (*P*<0.05).

The three identified proPOs in the transcriptome were designated as FcPPO1, FcPPO2 and FcPPO3, respectively. FcPPO1 shared almost the same amino acid sequences with a published proPO (FcproPO-p1, accession number: ABV60265) from *F. chinensis* [84], expect for the difference of two residues (Figure 9). This difference could be caused by existence of SNP in different individuals. FcPPO2 (KC138714) shared most of its amino acid sequence but 14 continuous residues and 5 other residues variances with a submitted proPO (FcproPO-p2, accession number: ACM61983) from *F. chinensis* (Figure 9). FcPPO2 also shared high similarity with FcPPO1, while FcPPO3 (KC138715) shared lower similarity with known proPO from *F. chinensis* (Figure 9), indicating that it was a new isoform in the Chinese shrimp. Phylogenic analysis revealed that proPO from shrimp, crayfish and crab were categorized into three groups. FcPPO1, FcPPO2 and proPOs from other penaeid shrimp, except for MjproPOb from *M. japonicus*, were clustered into group 1. proPOs from crayfish were clustered into group 2, while FcPPO3 and MjproPOb were clustered into group 3 with proPOs from crabs and a proPO from the fresh water prawn, *Macrobrachium rosenbergii* (Figure 10). Most of the identified proPOs were mainly detected in haemocytes [91]–[96], while MjproPOb and EsproPO were mainly synthesized in the hepatopancreas [97], [98]. qPCR analysis demonstrated a 3.4-fold up-regulation of FcPPO1, a 9-fold up-regulation of FcPPO2 and a 11-fold down-regulation of FcPPO3 in the AI shrimp compared to LI shrimp (Figure 11), in accordance with the transcriptome result. These data provided evidence for the first time that many genes in the proPO-activating cascade were stimulated by WSSV and indicated that the expression of different types of proPOs might be responded to different kinds of pathogens and regulated by distinct upstream mechanism.

### Regulation of WSSV infection by RNAi

In the transcriptome data, 8 DEGs were identified as key members of RNA interference (RNAi) pathway, including Argonaute 1, Argonaute 2 and Dicer 2 (Table S6). Primitively, researchers discovered that virus replication was significantly inhibited by dsRNA [99]–[103] and siRNA [104]–[106] of specific genes, revealing an RNAi mechanism against virus infection might exist in shrimp. Later, the genes encoding Argonaute, Dicer and other members of RNAi pathway were obtained in shrimp and evidence showed that they were involved in antiviral process via RNAi mechanism [107]–[110]. Therefore, more and more researchers deemed that application of RNAi machinery was an efficiently therapeutic method against virus infection [111]–[115] in shrimp aquaculture.

### Other AI stage responded cascades or genes

There were also plenty of other immune-related genes which were modified in the AI shrimp and listed in the Table S6. Many of these genes were previously identified as important members in host immunity against virus infection, including proteins with chitin binding domain (such as chitin binding PM protein, peritrophin , chitinase and cuticle protein in the present study), or C-type lectin domain (such as lectin, CRE-CLEC-202 protein, mannose-binding protein, protein CLEC-199 and hemolectin) [7], lipopolysaccharide and beta-1,3-glucan binding protein [116], scavenger receptor [117], transglutaminase [118], hemocyte homeostasis-associated protein [119] and so on. We would like to share their information in this paper with other researchers.

### Verification of transcriptome data by qPCR

We evaluated differential expression level of 14 candidate genes using qPCR to validate their expression patterns in the transcriptome data. We could see from the results (Table 3) that: 1) the up-regulation or down-regulation trends of 100% selected genes were consistent between the qPCR results and the transcriptome data, 2) the fold ratio between the qPCR results and the transcriptome data of 85.7% (12 in 14) selected genes was bigger than 0.5 and less than 2. The analysis showed that the transcriptome data can reflect an actual gene expression profiles in LI and AI shrimp.

**Table 3:** Comparison of relative gene expression fold in transcriptome data and qPCR results.

Gene name	Fold in transcriptome (AI/LI)	Fold of qPCR results (AI/LI)	Fold ratio (qPCR/transcriptome)
FcALF1	3.34	3.28	0.98
FcALF2	20.25	34.62	1.71
FcALF3	11.88	23.68	1.99
FcALF4	2.41	3.76	1.56
FcALF5	4.56	7.2	1.58
FcALF6	6.59	10.61	1.61
ALFFc	17.88	38.74	2.17
FcPPO1	3.66	3.43	0.94
FcPPO2	4.41	9	2.04
FcPPO3	0.08	0.09	1.13
Unigene22860_All	2.14	1.89	0.88
Unigene7673_All	2.53	2.41	0.95
Unigene15342_All	3.27	2.42	0.74
Unigene10139_All	4.5	3.01	0.67

## Conclusion

The present study focused on the difference of the shrimp transcriptome at WSSV LI stage and AI stage, aiming for discovery of underlying mechanisms involved in host defense against WSSV acute infection. Based on the present study, a lot of genes or pathways were found to be modified by WSSV acute infection in shrimp. It not only revealed many WSSV-responded pathways, such as expression of Toll and IMD regulated AMPs, Ras-regulated endocytosis and RNAi pathway, were positively modified by WSSV acute infection, but also uncovered that the anti-bacterially proPO-activating cascade were probably participated in antiviral process. In addition, this study provided a detailed data for identification of novel genes in shrimp, especially under the situation that the whole genome sequence of the shrimp was still not available.

## Supporting Information

Table S1 **Primers used for gene cloning and qPCR analysis.**
(DOCX)

Table S2 **Sequence information of proPOs and ALFs used in the present study.**
(DOCX)

Table S3 **Pathway analysis of DEGs and all unigenes.**
(XLSX)

Table S4 **The data of all the DEGs.**
(XLSX)

Table S5 **The data of 111 unigene sequeces identical to WSSV genome.**
(XLSX)

Table S6 **Detail information of 805 immune-related DEGs.**
(XLSX)
