# Supplementary material for: Transcriptome Analysis on Chinese Shrimp Fenneropenaeus chinensis during WSSV Acute Infection
Source: PLoS One. 2013 Mar 19;8(3):e58627. doi: 10.1371/journal.pone.0058627 (PMC3602427; doi:10.1371/journal.pone.0058627)
Supplement: Table S1 — Primers used for gene cloning and qPCR analysis. (DOCX) [file pone.0058627.s001.docx]

Table S1 Primers used for gene cloning and qPCR analysis

| Gene | Primer Name | Sequence (5'-3') | Annealing temperature (°C) |
| --- | --- | --- | --- |
| VP28 | VP28F1 | TGTGACCAAGACCATCGAA | 55.0 |
|  | VP28R1 | CCACACCTTGAATGTTCCC |  |
|  | VP28F2 | AAACCTCCGCATTCCTGTGA | 55.0 |
|  | VP28R2 | TCCGCATCTTCTTCCTTCAT |  |
| 18S rRNA | 18S-F | TATACGCTAGTGGAGCTGGAA | 56.0 |
|  | 18S-R | GGGGAGGTAGTGACGAAAAAT |  |
| ALFFc | FcALFqF | AAGCCTGGTGCTGGTGGTGT | 62.0 |
|  | FcALFqR | GAGTTCGGTTTTCTCGTTCCT |  |
| FcALF1 | FcALF1qF | ATGTCCTGCCCGTCCCTTAG | 59.2 |
|  | FcALF1qR | CCTCCGTTATCACGCCCTGT |  |
| FcALF2 | FcALF2qF | TGCGAGTGTCAGTCTTTAGC | 60.6 |
|  | FcALF2qR | CAATCCTGTGAGTTTGTCCG |  |
| FcALF3 | FcALF3qF | CAGGATTGTGGGAGACGGGA | 57.8 |
|  | FcALF3qR | CTGCTGCGTGTTTCGGCTAC |  |
| FcALF4 | FcALF4qF | ACGATGCGAGTCTTGGTCAG | 59.2 |
|  | FcALF4qR | CTCATCCGAGTGCCACAACC |  |
| FcALF5 | FcALF5qF | GCTTGTTGAGTCGCAGTCCT | 59.2 |
|  | FcALF5qR | GAGCCTGTCTTATGAAATCCTT |  |
| FcALF6 | FcALF6qF | AGACTTATGGAGGAACGGAGAC | 59.2 |
|  | FcALF6qR | ATTTGCTGCGGGTGTTGGAC |  |
| FcPPO1 | FcPPO1qF | TTGACCGTGACAGGAAAGGG | 59.2 |
|  | FcPPO1qR | CTGCCGGAGTTGCTGATAGT |  |
| FcPPO2 | FcPPO2qF | TGGCACCTCGTCTACCCTAT | 57.8 |
|  | FcPPO2qR | GCTGAAAGTCCTGCATTTTGG |  |
| FcPPO3 | FcPPO3qF | GGCGTAATGGGAGCCGTAGA | 59.2 |
|  | FcPPO3qR | GAAGAATCAAATGCCGAGGGA |  |
| Unigene22860_All | G1qF | GACACCCCAAGCAAGAAACG | 59.2 |
|  | G1qR | GAAAGCATCCTCAGCAATCCC |  |
| Unigene7673_All | G2qF | GCCGTCTGAACCTGCGTCTT | 59.2 |
|  | G2qR | CTCTGCTTTCCCTGCATCCC |  |
| Unigene15342_All | G3qF | GAATGGAATCTTGGCTGTAA | 55.0 |
|  | G3qR | TATGGGAGTAATGAGGTCAC |  |
| Unigene10139_All | G4qF | GTTTGGCAGCAATCCTCCTC | 57.0 |
|  | G4qR | AAGCCCAGTTGTCCAGTTCC |  |
