# Supplementary material for: Transcriptome Analysis on Chinese Shrimp Fenneropenaeus chinensis during WSSV Acute Infection
Source: PLoS One. 2013 Mar 19;8(3):e58627. doi: 10.1371/journal.pone.0058627 (PMC3602427; doi:10.1371/journal.pone.0058627)
Supplement: Table S2 — Sequence information of proPOs and ALFs used in the present study. (DOCX) [file pone.0058627.s002.docx]

Table S2 Sequence information of proPOs and ALFs used in the present study

| Name | Accession number | Species |
| --- | --- | --- |
| FcPPO1/ FcproPO-p1 | ABV60265 | *Fenneropenaeus chinensis* |
| FcproPO-p2 | ACM61983 | *Fenneropenaeus chinensis* |
| FcPPO2 | KC138714 | *Fenneropenaeus chinensis* |
| FcPPO3 | KC138715 | *Fenneropenaeus chinensis* |
| MjproPOb | BAL15152 | *Marsupenaeus japonicus* |
| LvproPO1 | ABX76968 | *Litopenaeus vannamei* |
| LvproPO2 | ABQ45957 | *Litopenaeus vannamei* |
| MjproPO | BAB70485 | *Marsupenaeus japonicus* |
| PmproPO | AAM77689 | *Penaeus monodon* |
| PsproPO | AAM77690 | *Penaeus semisulcatus* |
| HgproPO | CAE46724 | *Homarus gammarus* |
| HaproPO | AAT73697 | *Homarus americanus* |
| NnproPO | CCE46011 | *Nephrops norvegicus* |
| PlproPO | ADD70249 | *Panulirus longipes* |
| CqproPO | AFD61667 | *Cherax quadricarinatus* |
| ScproPO | ABD90511 | *Scylla serrata* |
| EsproPO | ABS19633 | *Eriocheir sinensis* |
| MrproPO | AAX48010 | *Macrobrachium rosenbergii* |
| FcALF1 | JX853774 | *Fenneropenaeus chinensis* |
| FcALF2 | JX853775 | *Fenneropenaeus chinensis* |
| FcALF3 | JX853776 | *Fenneropenaeus chinensis* |
| FcALF4 | JX853777 | *Fenneropenaeus chinensis* |
| FcALF5 | JX853778 | *Fenneropenaeus chinensis* |
| FcALF6 | JX853779 | *Fenneropenaeus chinensis* |
| ALFFc | AY859500 | *Fenneropenaeus chinensis* |
| ScALF | HQ638024 | *Scylla serrata* |
| SpALF | EF207786 | *Scylla paramamosain* |
| PtALF | GQ165621 | *Portunus trituberculatus* |
| PlALF | EF523760 | *Pacifastacus leniusculus* |
| FiALF | GU727863 | *Fenneropenaeus indicus* |
| FpALF | EF601051 | *Farfantepenaeus paulensis* |
| PmALF | EF523559 | *Penaeus monodon* |
| HaALF2 | EU625517 | *Homarus americanus* |
| LvALF | DQ208706 | *Litopenaeus vannamei* |
| MoALF | EU289220 | *Macrobrachium olfersii* |
| EsALF2 | GU014699 | *Eriocheir sinensis* |
